# Falling Trend in the Epidemiology of Gastric Cancer in Mississippi From 2003 to 2019: What Mississippi Got Right

**DOI:** 10.7759/cureus.31440

**Published:** 2022-11-13

**Authors:** Basil N Nduma, Solomon Ambe, Chukwuyem Ekhator, Ekokobe Fonkem

**Affiliations:** 1 Internal Medicine, Merit Health Wesley, Hattiesburg, USA; 2 Neurology, Baylor Scott & White Health, McKinney, USA; 3 Neuro-Oncology, New York Institute of Technology, College of Osteopathic Medicine, Old Westbury, USA; 4 Neuro-Oncology, Baylor Scott & White Health, Temple, USA

**Keywords:** incidence, epidemiology, mississippi, cancer registry, gastric cancer

## Abstract

Background: The aim of this study is to give insight into the falling trend in gastric cancer epidemiology in the state of Mississippi. The period in focus is between 2003 and 2019. The aim of this study is to uncover what the state got right and the implications for future healthcare.

Methodology: The data for this study was collected from the Mississippi state cancer registry. The geographic locations in focus are the state’s cancer coalition regions. The data is presented in tables and graphs, with descriptive and inferential statistical analysis.

Results: An assessment of the respective cancer coalition regions reveals a notable decline in gastric cancer incidence rates between 2003 and 2009. The areas where the state got right were found to include evaluation and surveillance, environmental, systems, and policy changes, treatment, survivorship, early detection, and prevention.

Conclusion: Given that the state is predominantly rural, it is recommended that additional innovative approaches are explored and implemented, including telemedicine implementation to foster real-time services regarding community health education and dissemination or messaging about actions such as gastric cancer screening and the needed environmental changes such as nutrition guideline adherence.

## Introduction

Over the last several decades, gastric cancer incidence and mortality have fallen dramatically, including in the United States (U.S.) and other parts of the world. However, this condition remains among the major issues facing the public health arena [1]. From the 2020 worldwide estimates by the Global Cancer Observatory (GLOBOCAN), gastric cancer accounted for close to 0.8 million deaths, reflecting about 7.7% of all deaths related to cancer [2]. In both genders combined, additional statistics suggest that gastric cancer ranks fourth among the leading causes of cancer-related deaths [3]. In 2020 also, nearly 1.1 million new cases were diagnosed in relation to stomach cancer, reflecting 5.6% of cancer cases in the entirety [4]. Evolving as one of the malignant tumors deemed the most lethal, gastric cancer has been associated with a 20% five-year survival rate [5,6]. In other studies, risk factors of gastric cancer have been documented. Some of them include radiation, obesity, tobacco, dietary factors, and Helicobacter pylori infection [7]. These studies highlight that to date, gastric cancer prevention can be achieved mostly through a reduction in exposure to risk factors and also early detection and screening initiatives.

In the first half of the 20th century in both Europe and the U.S., the leading cause of death due to malignant tumors was gastric cancer. In more recent decades, however, there has been a substantial decline in the rates in the majority of countries [8-10]. Evolving as a multifactorial disease, gastric cancer has been linked to specific attributes within the risk factors that were mentioned earlier. They include inherited predisposition, positive family history, gastroesophageal reflux disease, radiation, obesity, low physical activity, alcohol use, tobacco smoking, low fiber intake, fruit and vegetable consumption, and additional dietary factors in the form of high intake of smoked, salty food and low socioeconomic status [11,12]. Despite the strong decline, the condition remains burdensome. In particular, the majority of the risk factors are yet to be understood sufficiently. Hence, there is a need to have further research focused on this gap, with the aim of realizing more targeted and specific prevention measures. This paper gives insight into the falling trend of gastric cancer incidence in the state of Mississippi with a specific objective to uncover what the state got right and how the positive trend could be sustained into the future.

## Materials and methods

In Mississippi, it is at the state level that data concerning cancer incidence and mortality is collected, organized, and stored in the cancer registry for the purpose of future reference. This registry contains population-based data regarding population mortality, with gastric cancer data on disease incidence and mortality unexceptional. In particular, age-adjusted rates are reported. The implication is that in this current study, the main source of data is the state’s open-source cancer registry. The study sought to give insight into gastric cancer epidemiology with a specific focus on trends between 2003 and 2019. By giving insight into gastric cancer epidemiologic trends, the motivation is to highlight whether or not the rates were declining or promising and, if so, what factors were causing this trend. The additional motivation behind the study stems from the need to discern the intersection between social determinants of health and epidemiologic trends, areas worth sustaining, as well as opportunities worth exploiting and improving upon in the future.

The cancer registry allows an end-user to select and download open-source data for didactics. In this study, the specific options select from the open-source cancer registry include all cancer incidence, invasive cancer incidence, and cancer mortality. With this study’s focal area being gastric cancer epidemiology, the option chosen entailed all cancer incidence. In terms of geography, the Mississippi open-source cancer registry offers options such as data analysis by public health district, county, Delta or non-Delta regions, cancer coalition regions, rural or urban areas, and the Appalachian region. In addition, the geographical factor was then narrowed down to involve gastric cancer by cancer coalition region. The cancer registry also allows an end user to select the cancer site to focus upon. In this case, the stomach was selected as the cancer site because the study’s focus was on the central subject of gastric cancer. Additional navigation of the registry paves the way for the user to select the starting year and the ending year to ensure the timeframe being focused upon is specified. With age-adjusted rates on the focus, the starting year that was chosen was 2003 while the ending year was 2019. The registry provides additional room for the user to select the sex being focused upon and, in this case, all sexes were selected, implying both male and female populations had their gastric cancer used in the context of Mississippi. Relative to the factor of race or ethnicity, options include Black, White, or all, and the choice that was arrived upon was all races or ethnic groups, implying Mississippi’s general population formed the focal demographic area.

Upon collecting, organizing, and presenting, we analyzed the open-source data by implementing descriptive and inferential statistics as presented in simple data tables and graphs approach. The goal is that graphical presentation and the tabulation approach aid in descriptive statistics while inferential statistics complement the descriptive statistical outcomes. In the end, the epidemiology of gastric cancer between 2003 and 2019 was determined from a trend perspective before culminating in the discussion of the interplay between the results that were obtained and social determinants of health in the state, aiming to discern what the state got right within the period in case of declining trends and the implications for the future of the healthcare sector and gastric cancer incidence in the state.

The Data on the Mississippi cancer registry contains reportable conditions such as cancer-based on the International Classification of Disease, Ninth Revision, Clinical Modification (ICD-9-CM) codes, and ICD-10-CM codes. The description of these cancers does not change often but the ICD-10-CM codes may change annually. Per the ICD-10-CM Diagnosis code 2022/2023, the Malignant neoplasm of the stomach which is based on the histologic description and morphology of the tumor is code C16. Of note, therefore, is that all histologic types of cancers as long as they meet the definition of malignant neoplasm of the stomach are included in this definition. These include but are not limited to the following: all subtypes of adenocarcinomas of the stomach, signet ring cell carcinoma, mixed carcinoma, squamous carcinoma, lymphoma, choriocarcinoma, parietal cell carcinoma, malignant rhabdoid tumor, mucoepidermoid carcinoma, neuroendocrine carcinomas, etc. The unit used for the age-adjusted cancer rates in Mississippi is per 100,000.

## Results

**Figure 1:**
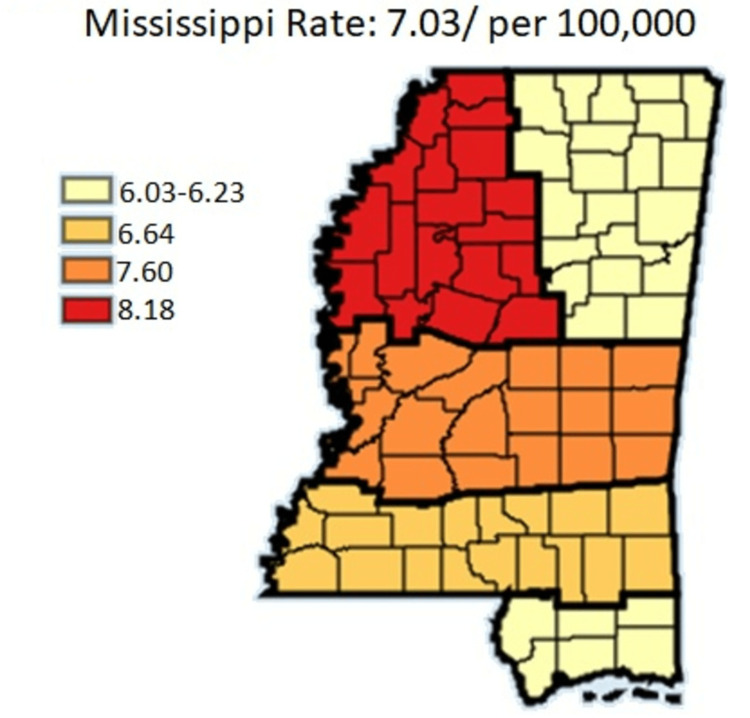
Gastric cancer incidence per cancer coalition regions for Mississippi state Age-adjusted to the 2000 U.S. Standard Million Population. The population estimates for 2005 are adjusted to account for population shifts due to Hurricane Katrina (www.seer.cancer.gov/popdata/)

This section presents the results that were obtained, with the source of data being the cancer registry in Mississippi. Comparing the five cancer coalition regions between the years 2003 and 2019 shows that Delta regional coalition had the worst gastric cancer incidence rates compared to the Coastal regional coalition regions which has the least gastric cancer incidence rate between 2003 and 2019. Table 1 indicates values for the age-adjusted rate per 100,000 depicting a comparative analysis of the five cancer coalition regions. Figure 1 outlines the gastric cancer incidence per cancer coalition region.

Table 1 shows the tabular representation of gastric cancer incidence between 2003 and 2019. From Table 1, the incidence rate was highest in Delta region, followed by Central, Southern, Northeast, and Coastal regional coalitions, respectively. In that order, the age-adjusted rates as established from the cancer registry for the regional coalitions were 8.18, 7.60, 6.64, 6.23, and 6.03 per 100,000.

**Table 1:** Tabular representation of gastric cancer incidence between 2003 and 2019

Region	Population at Risk	Cases	Crude Rate	Age-adjusted Rate	95% Confidence Interval
Delta Regional Coalition	9025129	773	8.56	8.18	[7.60, 8.78]
Central Regional Coalition	14827121	1201	8.10	7.60	[7.17, 8.05]
Southern Regional Coalition	8058873	603	7.48	6.64	[6.11, 7.19]
Northeast Regional Coalition	10295688	716	6.95	6.23	[5.78, 6.71]
Coastal Regional Coalition	8015346	535	6.67	‘6.03	[5.52, 6.57]

**Figure 2:**
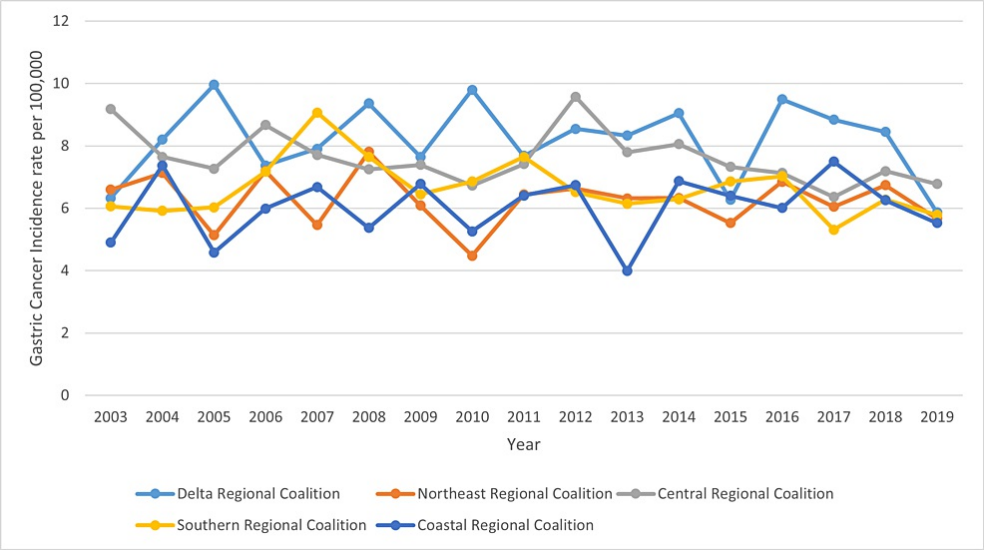
Trend in gastric cancer incidence rate in Mississippi by cancer coalition regions from 2003 to 2019 The unit or denominator for the gastric cancer incidence rate measured is per 100,000

Delta regional coalition (Figure 2) despite rating worst among the cancer regional coalitions in Mississippi, experienced a notable decline in gastric cancer incidence, whereas there was a sharp increase in disease incidence between 2003 and 2005, the years stretching from 2006 to 2014 experienced fluctuations involving increasing and decreasing incidence rates, and it was only between 2013 and 2014 and 2014 and 2015 that there were sharper fluctuations involving increasing and declining rates, respectively. Beyond 2016, this cancer coalition region witnessed a decline in disease incidence, with the year 2019 experiencing the lowest trend in the 16-year period of this study.

Compared to the case of Delta, the situation in the Central regional coalition as outlined in Figure 2 above is even more promising. The upward trends were only in the period between 2005 and 2006 and also between 2010 and 2012, with the rest of the phase witnessing a significant decline in gastric cancer incidence rates. The periods in which declines in disease incidence rates were significant included the phase from 2006 and 2010 and the range between 2012 and 2017.

In the Southern regional coalition in Figure 2, a sharp increase in the incidence rate was witnessed between 2005 and 2007, with the preceding two years witnessing no significant change in the epidemiologic depiction. Beyond 2007, however, this region experienced consistently lower values, ranging from 5.31 as the lowest value in 2017 to 7.65 as the highest value in 2011 as per data comparisons beyond the year 2011. What is worth noting, however, is that the rate between 2017 and 2018 was similar in trend to the case of Central in which the incidence rate increased slightly before declining between 2018 and 2019 in both regional coalitions.

Whereas values for disease incidence rate in the Northeast regional coalition illustrated in Figure 2 were consistently lower than the situation in Delta, Southern, and Central, fluctuations in epidemiologic trends were more notable. Also, similar to the case of Central and Southern, there was an increase in the incidence rate between 2017 and 2018 before declining between 2018 and 2019. The Northeast region recorded its lowest incidence of 4.48 as the age-adjusted rate in 2010. The overall trend was a decline in the incidence rate in the studied period.

**Figure 3:**
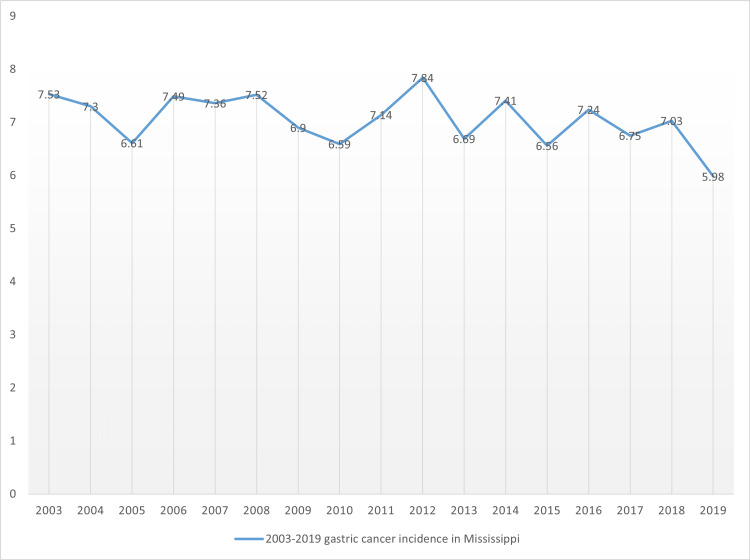
Statewide age-adjusted gastric cancer incidence rate in Mississippi state

Out of the five cancer coalition regions, it was the Coastal regional coalition illustrated in Figure 2 that showed the lowest incidence rate reported, with the year exhibiting the least age-adjusted rate being 2013 in which a value of 3.99 was reported. Also, this region experienced notable fluctuations in trends in disease incidence. The highest incidence rate was recorded in 2017 at 7.5, but the value also proved the lowest compared to the highest incidence rate values per year for the first four regional coalitions. Hence, the highest age-adjusted value in Costal remained the lowest of all the highest values based on regional comparisons. Conversely, the lowest age-adjusted value for Costal proved the lowest of all the least incidence rates per year as per the comparison of the outcomes among the five regional coalitions. When statewide data are considered in Figure 3, the lowest age-adjusted rate was 5.98 in 2019 while the highest rate occurred in 2012, which was 7.84 per 100,000.

**Figure 4:**
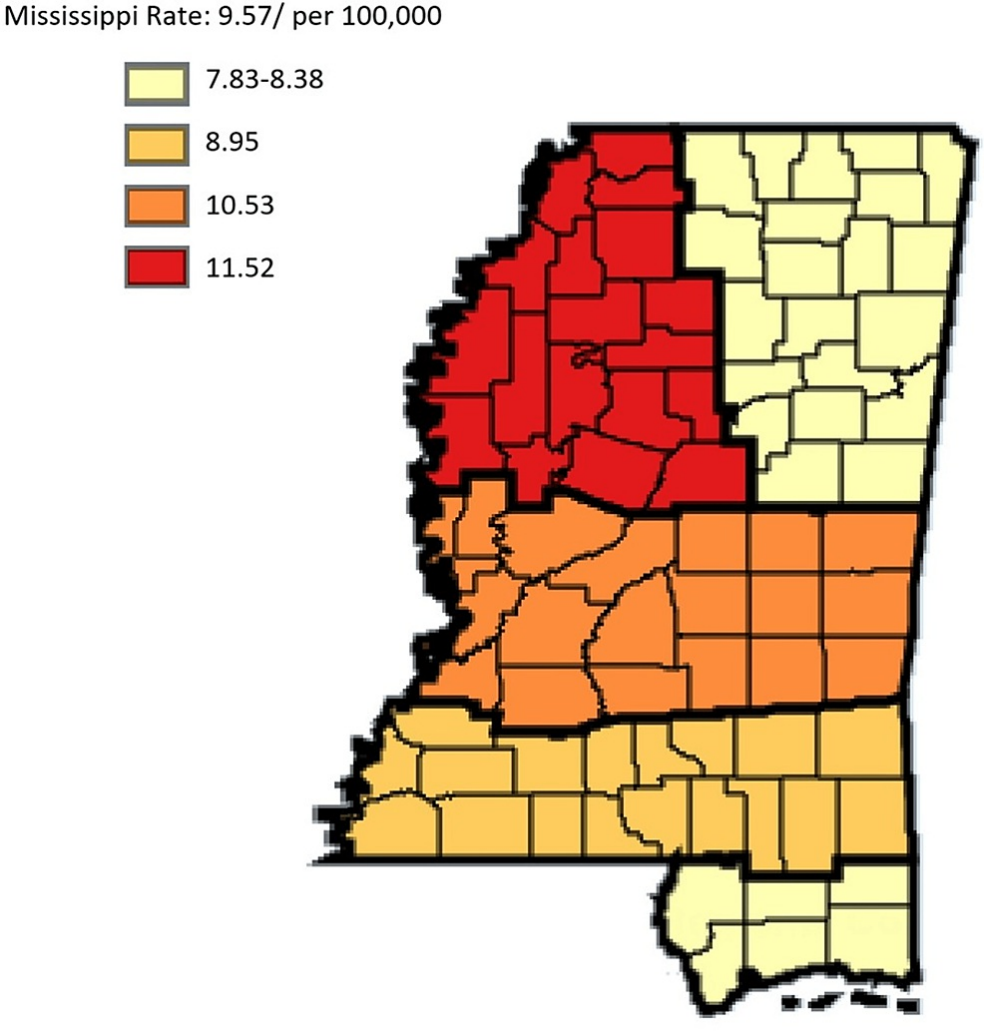
Incidence of gastric cancer in male residents per cancer coalition regions in Mississippi state from 2003 to 2019 Age-adjusted to the 2000 U.S. Standard Million population. The population estimates for 2005 are adjusted to account for population shifts due to hurricane Katrina (www.seer.cancer.gov/popdata/)

**Figure 5:**
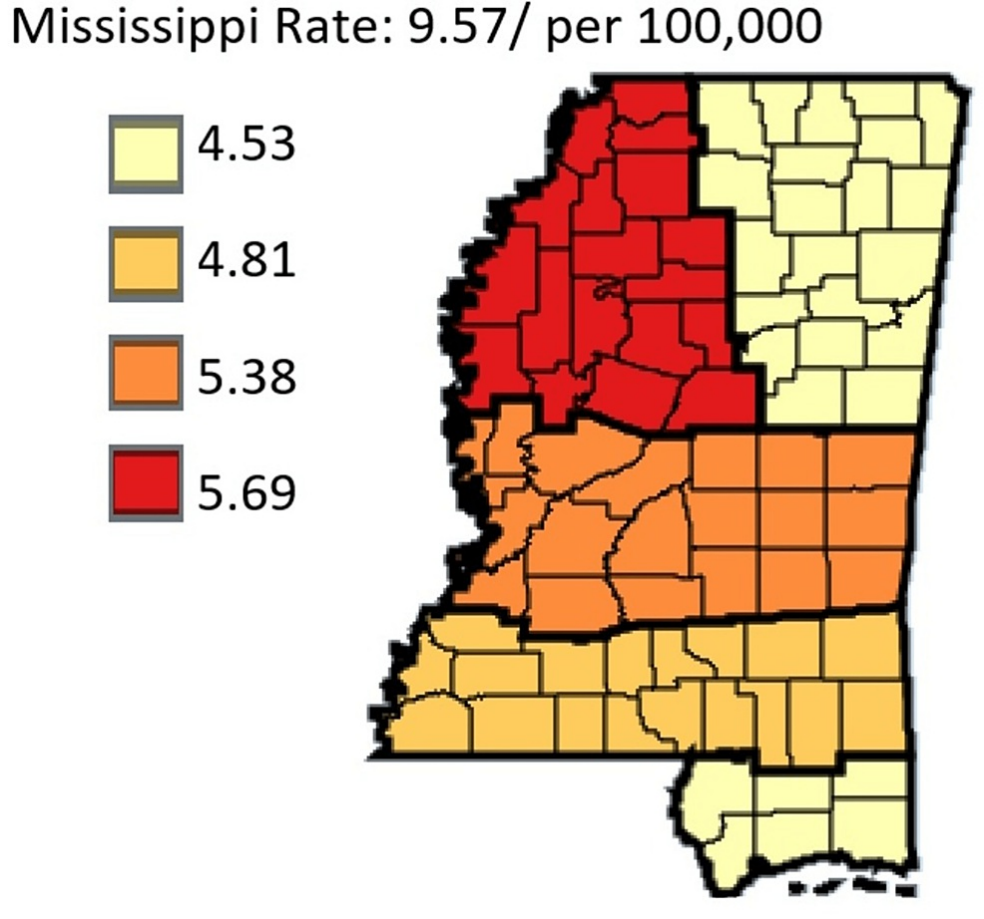
Incidence of gastric cancer in female residents per cancer coalition regions in Mississippi state from 2003 to 2019 Age-adjusted to the 2000 U.S. Standard Million population. The population estimates for 2005 are adjusted to account for population shifts due to hurricane Katrina (www.seer.cancer.gov/popdata/)

From 2013 to 2019, the incidence rates were consistently lower. Also, fluctuations from one year to another were evident, the graph was found to be relatively flatter, with steeper fluctuations only observed between 2004 and 2006 and also between 2010 and 2012. In this study, the trends of gastric cancer in males and females (Figures 4, 5) were also examined. Figure 4 outlines the incidence of gastric cancer in male residents per cancer coalition regions in Mississippi state.

Figure 5 outlines the incidence of gastric cancer in female residents per cancer coalition regions in Mississippi state.

**Figure 6:**
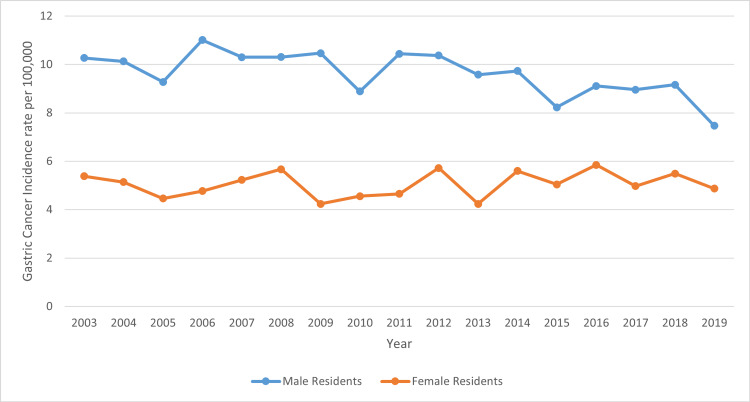
Comparing trends in gastric cancer incidence rate in Mississippi, male vs female residents from 2003 to 2019

Based on Figure 6, in the state of Mississippi, females have a lower incidence rate per 100,000 of gastric cancer at the rate of 5.05 compared to males who had an incidence rate of 9.57.

**Figure 7:**
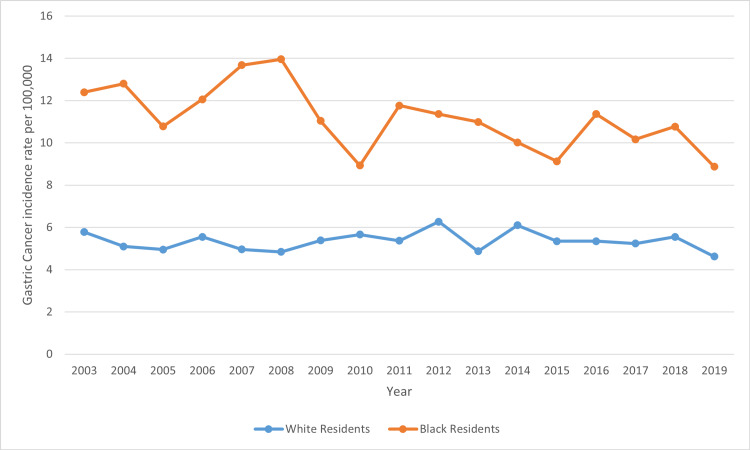
Comparing trends in gastric cancer incidence rate in Mississippi, White versus Black residents from 2003 to 2019

This study also demonstrated the incidence rates of gastric cancer in white versus black residents in Mississippi state. White residents had a lower incidence of gastric cancer in Mississippi at a rate of 5.33 compared to black residents who had a higher rate of 11.10 per 100,000 (Figure 7). The incidence rates of gastric cancer in the regional coalition regions by ethnicity and sex show that Delta regional coalition consistently had the highest incidence rate, and the Northeast regional coalition consistently had the lowest incidence rate along with the coastal regional coalition for the most part of the analysis.

**Figure 8:**
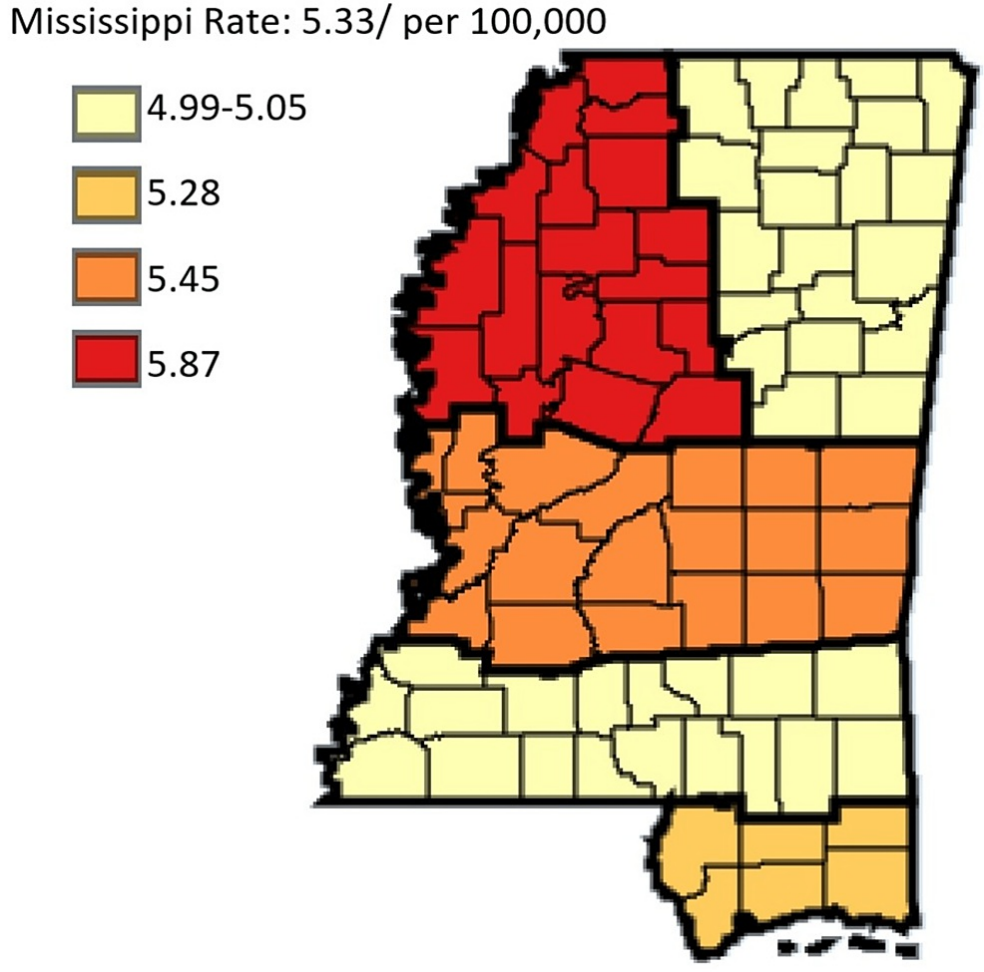
Gastric cancer incidence of white residents per cancer coalition regions in Mississippi state Age-adjusted to the 2000 U.S. Standard Million population. The population estimates for 2005 are adjusted to account for population shifts due to hurricane Katrina (www.seer.cancer.gov/popdata/)

Figure 8 outlines the Gastric Cancer incidence of white residents per cancer coalition regions in Mississippi state.

**Figure 9:**
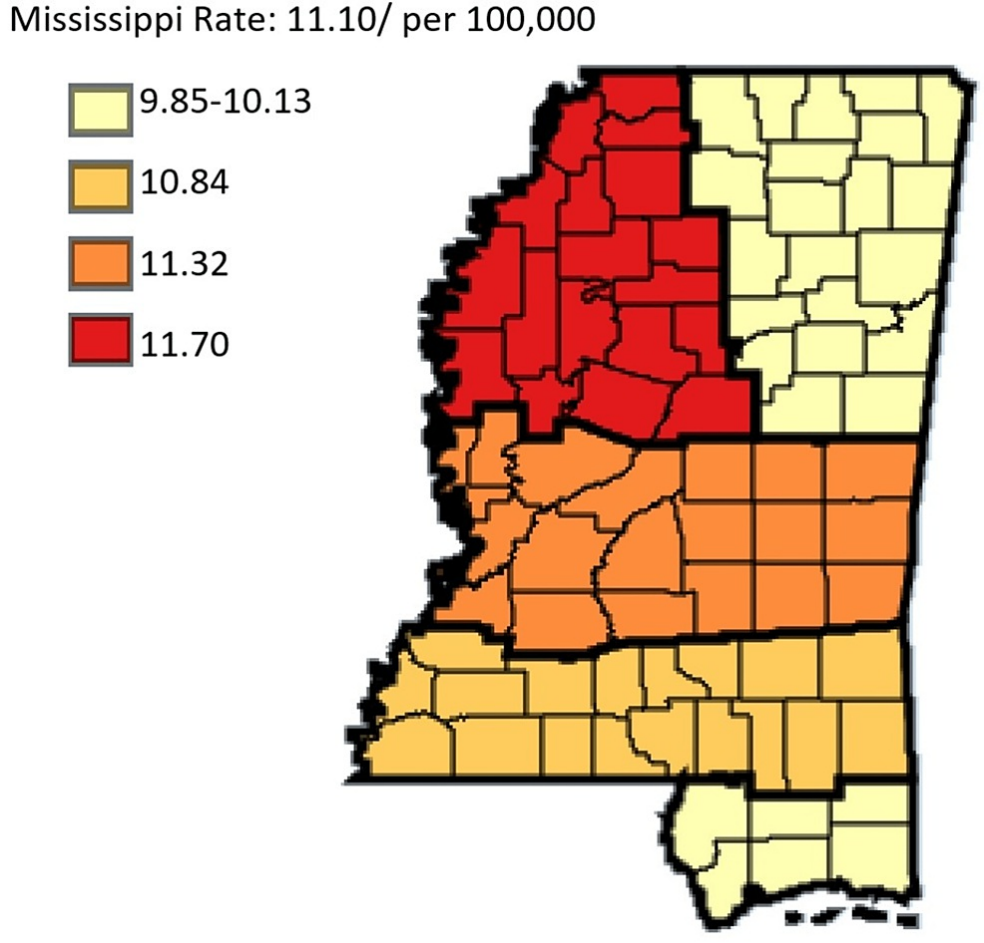
Gastric Cancer incidence of black residents per cancer coalition regions in Mississippi state Age-adjusted to the 2000 U.S. Standard Million population. The population estimates for 2005 are adjusted to account for population shifts due to hurricane Katrina (www.seer.cancer.gov/popdata/)

Figure 9 outlines the gastric cancer incidence of black residents per cancer coalition regions in Mississippi state.

The trend line graphs from Figures 6, 7 above depict the incidence rates in Mississippi, statewide. This shows the trend of gastric cancer in male versus female residents and white versus black residents. Data for other ethnic groups are not captured in the Mississippi state cancer registry database because other ethnic groups outside of white and black residents form a very small percentage of the population. In addition, the trend line graph from Figure 7 shows that the incidence rate of gastric cancer in black residents is higher than the incidence rate for white residents. Also, the incidence rates for male residents are higher than the incidence rate for female residents. In all cases, the incident rates overall were on the decline during the period captured by this study.

## Discussion

The falling trends in the incidence of gastric cancer during the studied period in Mississippi can be explained through the Mississippi Partnership for Comprehensive Cancer Control (MP3C). MP3C is a group of individuals and organizations united to fight cancer in Mississippi via preventive measures, surveillance, early detection, and treatment. Through this program, Mississippi has witnessed a coordinated and collaborative efforts aimed at reducing gastric and other cancer risk factors at the environmental, systems, and policy change levels within government and faith settings, worksites, communities, and schools. Specifically, the MP3C leadership has been engaged in identifying a minimum of three priority areas that could be targeted by a state-wide agenda in a quest to realize environmental, systems, and policy change at the local, regional, and state levels [13,14]. Also, there has been technical assistance, training, and education linked to propose environmental and policy change, as well as the promotion of the adoption of best practice or evidence-based interventions to address pollution and tobacco-free environments, nutrition standards, and physical activity at the municipal, county, and state levels [15].

In relation to the attribute of early detection as another area where Mississippi got right, assets and resources needed for gastric cancer have been defined and established in such a way that the MP3C has partnered with a minimum of one community-based organization in each regional coalition, eventually ensuring increased knowledge across genders regarding the criticality of gastric cancer screening [16]. A specific action has been encouraged by MP3C membership to ensure engagement and fostering of partnerships to have messaging and health education about gastric cancer delivered among targeted communities. Similarly, the MP3C has collaborated with partners to have current cancer education coined in a manner that can be deemed culturally appropriate relative to the targeted communities [17]. Similarly, the MP3C has collaborated with community organizations and leaders to ensure an increase in the number of community health workers and patient navigators, who have been trained to ensure care access increases. Community awareness concerning resources for no-cost or low-cost cancer screening services has been promoted, with underserved neighborhoods particularly targeted in Mississippi [17,18]. The initiatives have been completed by collaboration with community and faith organizations, translating to culturally appropriate and comprehensive gastric cancer education. Still, the number of health and wellness initiatives has increased in Mississippi, ensuring early detection is increased by as high as 25% [18]. Across the state, therefore, the MP3C has conducted environmental scans to uncover current ministerial alliances, faith-related associations and groups, and health ministries. In turn, resources, tools, training, and education about early gastric cancer detection have been provided, especially among the uninsured, underinsured, underserved, and minority populations [18,19]. The developments have been complemented by regional cancer screening referral lists that have been developed.

It is also in Mississippi and courtesy of the MP3C that clinical trials have been conducted relative to gastric cancer incidence and prevention. In particular, the MP3C has promoted awareness and participation in gastric cancer clinical trials. In addition, there has been the promotion of organizational and public policy development in a manner aimed at encouraging participation in and access to community-level clinical trials. Particularly, the MP3C has collaborated with cancer advocacy institutions to ensure that insurance payers in the context of Mississippi are supported in covering standard care costs that come with treatment in the course of clinical trial implementation for gastric cancer [19]. Still, with gastric cancer on the focus, clinical trial participants have been recruited to serve as volunteer spokespeople; ensuring education is extended to policymakers concerning benefits and the value of clinical research access support at the local level, including the criticality of ensuring that availability of enough health coverage costs linked to the clinical trials that are implemented [20]. Educational materials that indicate patient stories have also been developed and disseminated to policymakers in Mississippi.

Another focal area that explains the decline in gastric cancer rates and reflects where Mississippi got right entails survivorship. Here, systems serving gastric cancer survivors have been identified throughout the state’s respective regions. In turn, partnerships with regional organizations have been increased to ensure Stanford Chronic Disease Self-Management Program training is conducted for gastric cancer survivors in the respective regions, an effort that has been complemented by the monitoring, tracking, and provision of technical assistance to systems that conduct the training. Gastric cancer survivorship information has also been gathered and disseminated through MP3C and associated websites. At the same time, there has been multi-disciplinary team development on the part of healthcare providers for gastric cancer patients through the quality improvement initiative in Mississippi, a move that has contributed significantly to team-based care effort improvement for survivors [12,13]. Additional groups that have been engaged include pediatric oncologists, schools, and professionals serving in the MP3C to ensure programs for gastric cancer survivors are planned and implemented.

Regarding the attribute of treatment, gastric cancer care providers in Mississippi have been involved in statewide gastric cancer clinical trials, with a specific emphasis on organizations serving the underserved and minorities to ensure they are represented adequately in studies. Groups experiencing health inequities, minority health community organizations, and communities have been supported and engaged in the identification and solution of issues related to access to care. For providers and patients in rural or remote locations, partnerships have been promoted and supported to ensure access to specialty services for gastric cancer is facilitated, including situations prompting the use of telemedicine. For medically underserved groups, patient access programs that offer gastric cancer medications (chemotherapy) have been promoted through a partnership with public health programs such as gastric cancer-related organizations, with the outcome being an increase in the utilization of treatment programs [20].

There have also been environmental, system, and policy changes in Mississippi, with a particular focus on gastric cancer prevention. For instance, there has been an increase in the number of churches and municipalities adopting the environmental, system, and policy changes in terms of nutrition guidelines, shared-use agreements, and smoke-free air. To ensure a smoke-free environment is implemented, the MP3C has strengthened collaborations through a partnership with municipalities and Mississippi’s Office of Tobacco Control. Additionally, technical assistance to faith-based institutions has been offered, with similar services extended to wellness ministries [11]. Community access points in the form of churches have also increased to ensure gastric cancer training and education, with Congregational Health Nurses also trained on how to implement gastric cancer control programs, efforts, or initiatives [19].

Lastly, there has been gastric cancer evaluation and surveillance in Mississippi. Specifically, the number of evaluation methodologies has been increased, especially those charged with evaluating annual objective implementation. Thus, data-driven resources have been created, which have then been utilized by grantees to have gastric cancer addressed in the respective coalition regions. Relative to the evaluation methods also, training materials have been revised and disseminated, with the number of surveillance methods and data for discerning the burden of gastric cancer maintained, including local interventions, vital statistics, cancer registry, and hospital discharge data.

The data demonstrate that in Mississippi, statewide control and prevention of gastric cancer has been relatively organized. The key actions that explain the decline in gastric cancer incidence rate between 2003 and 2019, which reflect where Mississippi got right, include evaluation and surveillance, environmental, systems, and policy changes, treatment, survivorship, early detection, and prevention. With the state being predominantly rural, however, it is recommended that additional effort be directed at the factor of access to care by enhancing the attribute of telemedicine, which tends to be real-time. The Telemedicine industry is growing rapidly in the USA today and it provides access to both treatment and prevention services. Hence, even as populations are sensitized about the needed environmental changes and the criticality of participating in clinical trials and tracking surveillance data, underserved neighborhoods in remote locations require additional effort to ensure that gastric cancer treatment and prevention services are brought closer to the people and telemedicine could be help increase this access.

Assessment and limitation of the study

In the search process for this study, the gastric cancer incidence data from the Mississippi cancer registry site was limited to 2003 through 2019. This signifies the omission of data that was available prior to 2003 and beyond 2019. Also, the individual sociodemographic variables are not a focus of this study. Further studies are needed to investigate the distribution of sociodemographic variables with decreasing trend of gastric cancer incidence in the state of Mississippi.

## Conclusions

In summary, this paper focused on declining trends in gastric cancer incidence rates in Mississippi. With data drawn from the open-source Mississippi cancer registry, an assessment of the respective cancer coalition regions demonstrated a notable decline in the rates between 2003 and 2009. Areas where the state got right include evaluation and surveillance, environmental, systems, and policy changes, treatment, survivorship, early detection, and prevention. Given that the state is predominantly rural, it is recommended that additional innovative approaches are explored and implemented, including telemedicine implementation to foster real-time services regarding community health education and dissemination or messaging about actions such as gastric cancer screening and the needed environmental changes such as nutrition guideline adherence.
